# Returning to work after maternity leave: a systematic literature review

**DOI:** 10.1007/s00737-024-01464-y

**Published:** 2024-04-05

**Authors:** Isabella Giulia Franzoi, Maria Domenica Sauta, Alessandra De Luca, Antonella Granieri

**Affiliations:** Department of Psychology, University of Turin, Via Verdi 10, Turin, 10124 Italy

**Keywords:** Maternity leave, Returning to work, Mental health, Job related well-being, Work-life balance

## Abstract

**Purpose:**

Working women often experience difficulties associated with balancing family and career, particularly if they choose to have children. This systematic literature review aimed at investigating women’s experience in returning to work after maternity leave.

**Methods:**

The review was conducted using the Preferred Reporting Items for Systematic Reviews and Meta-Analyses. The literature search led to the identification of 52 articles, which underwent data extraction and qualitative analysis.

**Results:**

Results were organized in 5 categories: (1) Work-life balance; (2) Women’s mental and physical health; (3) Job-related wellbeing and working experience; (4) Breastfeeding. Women’s both mental and physical health seem connected to a longer maternity leave and a greater coworkers’ and supervisors’ support. Returning to work seems to constitute one of the most important barriers for exclusive breastfeeding or breastfeeding continuation. A shorter duration of maternity leave, a higher workload and the lack of occupational policies supporting breastfeeding seem to be hindering factors. Partner and family support, and the opportunity for fathers to work under a flextime system after childbirth seem to increase both breastfeeding initiation and duration. Women who continue breastfeeding after returning to work seem to experience more family-to-work conflict and overload.

**Conclusions:**

This paper show that there are still many understudied aspects in exploring women’s experience of returning to work after maternity leave. This represents an important gap in the literature, since returning to work represents a particularly critical time in women’s personal and occupational life, in which challenges and barriers may arise, potentially affecting their experience in the immediate future and years to come.

**Supplementary Information:**

The online version contains supplementary material available at 10.1007/s00737-024-01464-y.

## Introduction

In recent decades, the importance of women’s employment in the world of work has been increasingly recognized and valued, thanks in part to a growing number of studies showing that women’s work has positive consequences for a Country’s development and growth, not only in employment, but also in the economic, financial, and social spheres (Davidson and Burke 2016; Boca et al. 2020; Hakim 2016; Kabeer 2021).

This has led, at least in high-income Countries, to an increasing adoption of policies that support women’s work and better work-life balance, understood as the possibility of being meaningfully involved in both one’s work life and one’s personal life, being able to balance the demands coming from both of these spheres and satisfactorily living one’s role within the work context, the couple, the family, and society more generally (Wiens et al. 2023).

The introduction of paid maternity leave is one of the policies that has been most conducive to women’s employment we have (Olivetti and Petrongolo 2017): by the end of the 20th century, most high-income countries had enacted national laws regulating paid parental leave, while Australia, New Zealand, Switzerland, and the United Kingdom did not introduce such legislation until the beginning of the 21st century (Rossin-Slater 2017). The United States remains the only Organization for Economic Co-operation and Development (OECD) country without a federal provision for paid parental leave, while even in other OECD countries even today such leave is not universally available to self-employed, domestic, part-time, and casual workers (Girsberger et al. 2023).

However, while parental leave provides job protection, it does not protect against the negative impact of such interruptions on career progression, pay, and other aspects of working life (Blair et al. 2016; Fitzenberger et al. 2016; Lalive et al. 2014; Twamley and Schober 2019). Indeed, regulations alone are not sufficient to ensure a concrete and effective situation of equal opportunities and equal treatment between women’s employment and men’s employment: to date, significant differences between men and women remain at the level of career prospects, professional qualifications, entrepreneurial training, and equal pay, in addition to the fact that the burden associated with managing children, home and family life often remains heavily unbalanced on women (European Institute for Gender Equality 2019; Kleven and Landais 2017).

While having an occupation seems to have positive effects for women in terms of their physical and mental health, social support, and the economic resources available to them (Coulson et al. 2012), often the difficulties associated with balancing family and career can add an extra burden to the postpartum period, which is already particularly challenging for mothers’ somato-psychic balance (Chung and Van der Horst 2018; Gragnano et al. 2020; Hjálmsdóttir & Bjarnadóttir, 2021).

In particular, returning to work after a period of maternity leave is a key aspect in the personal and professional trajectory of women, which often becomes a challenge in several respects. Indeed, for women, is necessary to re-signify their roles as mothers and workers, their aspirations in career progression, and to organize and manage the time to devote to both childcare and work, with a potential negative impact on mental health, work-related satisfaction, and even couples (Cheng et al. 2016; Lucia-Casademunt et al. 2018; Powell and Karraker 2019; Røsand et al. 2011).

Women’s mental health and well-being concern an extremely delicate sphere in the pre, during, and post-pregnancy phases. In the postpartum, psychological distress can emerge at various levels (Chauhan et al. 2022; Steen and Francisco 2019; Makregiorgos et al. 2013) that are often linked to risk behaviors such as smoking, substance and alcohol use that can lead to a low quality of life for mother and child (Bedaso et al. 2021). As a result, children’s growth and development can also be negatively affected (Wallwiener et al. 2019). Indeed, the literature points out that children of mothers with psychological distress are at risk for poor health, developmental and behavioral problems, and emotional problems (Ali et al. 2013).

Therefore, the aim of this paper is to investigate women’s experience of returning to work following maternity leave through a systematic review of the literature published on the topic over the past 10 years.

## Materials and methods

### Search strategy

This systematic review was conducted following the Preferred Reporting Items for Systematic Reviews and Meta-Analyses (PRISMA) 2020 guidelines (Page et al. 2021).

Studies were identified by searching the following electronic databases: PsycInfo/PsycArtcicles, PubMed, Scopus, and Web of Science. We used a combination of the following keywords: “maternity leave” AND “return to work” OR “returning to work” OR “return-to-work” OR “back at work” OR “back to work” OR “end of the maternity leave” OR “end of leave” OR “end of maternity leave”.

The search fields were as follows: (1) TX-All Text for PsycInfo/PsycArticles and (2) all fields for every other database. We also limited our search to: (1) Source: Journal and Doc type: Article for Scopus; (2) Document Types: Article for Web of Science; (3) Species: Humans for PubMed and (4) Population group: Human, Document Type: Journal Article for PsycInfo/PsycArtcicles.

To collect the most recent data, the search was limited to journal articles published since January 2013 to December 2022. We decided to include only the most recent literature in this systematic review. As in other systematic reviews, therefore, only the last 10 years were considered (Speroni et al. 2023; Kriakous et al. 2021; Pollard and Lee 2003).

Moreover, only papers written in English were included, recognizing that this might lead to important results being overlooked and limited corroboration of reported data. Articles were retrieved on April 1, 2023.

### Selection criteria

The progressive exclusion of articles was performed by 3 authors of this paper [IGF, ADL] who read the title, the abstract, and the full text. In case of disagreement, a third author [MDS] was consulted.

Progressive exclusion was performed reading the title, abstract, and full text.

The inclusion criteria were as follows:

1. quantitative and qualitative studies;
2. research explicitly referring to the experience of returning back to work after maternity leave;
3. research focusing on the experience of women who were working before their pregnancy and/or who return to work within one year of their child’s birth;
4. research focused on formal workers;
5. research based on the perspective of women involved.

The exclusion criteria were as follows:

1. research not presenting original data (e.g., commentaries, systematic reviews);
2. research focusing on the experience of working women while pregnant or during the maternity leave;
3. research focusing on the choice of whether or not to return to work following maternity leave and on the correlates of this choice;
4. research focusing on women working after having a child, but not explicitly focusing on the experience of returning back to work;
5. research focusing on women who weren’t working before their pregnancy and/or who return to work more than one year after their child’s birth;
6. research focused on informal women workers;
7. research referring to the experiences of fathers;
8. research based on the perspective of fathers, health professionals, etc.

**Fig. 1:**
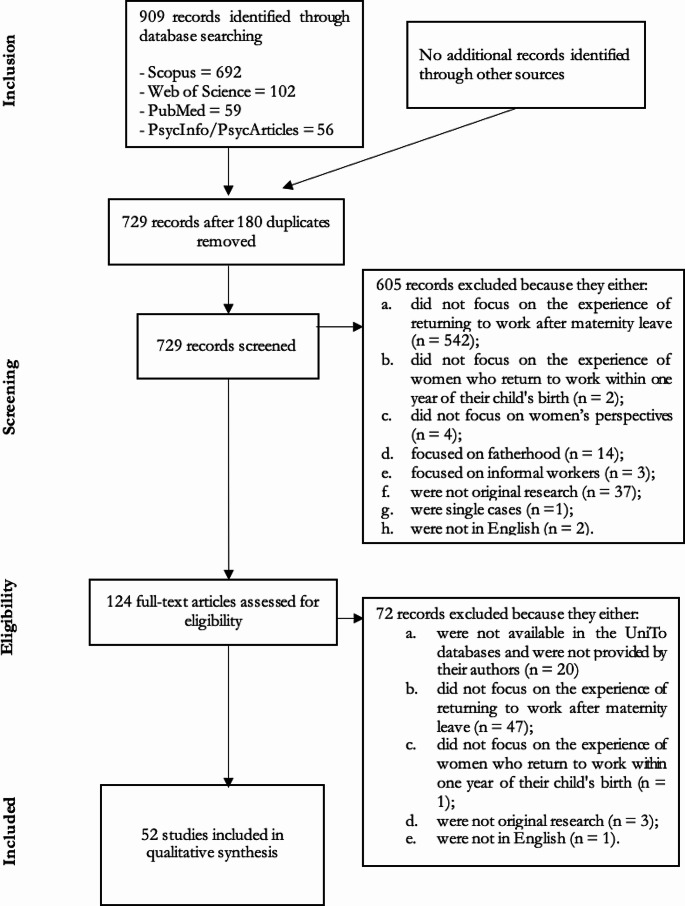
Flow of information through the different phases of the study selection process

A list of excluded studies, including level (title, abstract or full text) and reasons for exclusion, was retained. The entire procedure is displayed in Fig. 1.

### Data analysis

Data analysis was conducted using a standardized data extraction form covering (a) general study details (publication year, authors, title, publication source); (b) study type; (c) sample characteristics (e.g., age); (d) measures; I results; (f) country of the research (Supplementary material, Table 1).

All papers included in the systematic review were subjected to rigorous appraisal using the Critical Appraisal Checklist for Case Control, Cohort, and Cross-Sectional studies (Moola et al. 2020). The Joanna Briggs Institute offers well-established reliability and validity tests to assess the risk of bias in studies (Moola et al. 2020; https://jbi.global/critical-appraisal-tools).Three authors of this paper [IFG, MDS] independently evaluated the quality of each study. In case of disagreement, a third author [AG] was consulted. After the evaluation using JBI’s Critical Appraisal Checklist for, cross-sectional, cohort, and qualitative studies, all articles received an acceptable quality appraisal to be included in the current review. Articles were evaluated based on the following criteria: LOW risk of bias studies with more than 70% “yes” score; MODERATE risk of bias studies with 50–69% “yes” score; and HIGH risk of bias studies with less than 49% “yes” score. As recommended by the JBI reviewers’ manual, all decisions regarding the scoring system and cut-off points were approved by all reviewers before the start of the critical appraisal process.

## Results

The electronic database search identified 909 articles. After 180 duplicates were removed, 729 articles remained. Of these, 605 articles were excluded based on title and abstract, and further 72 articles were excluded based on full-text evaluation (Fig. 1). This left 52 articles for data extraction and qualitative analysis.

### Study characteristics

**Fig. 2:**
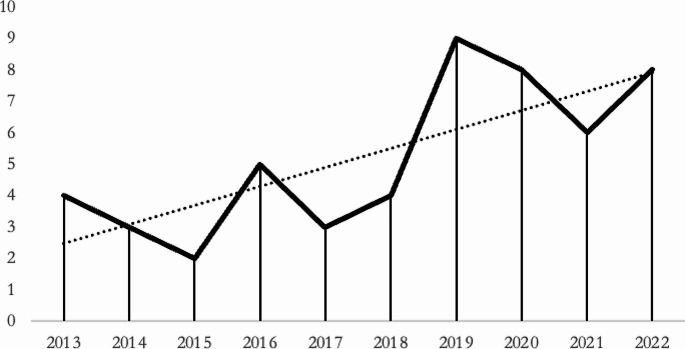
Trend over the years

The number of articles published in the years considered for the systematic literature review shows a growing scientific interest in the topic, with 31 (59.62%) articles published in the 2019–2022 time range (Fig. 2).

Regarding the geographical distribution of the studies, 12 (23.08%) were from North America and particularly the USA, 18 (34.62%) from East Asia and Pacific, 7 (13.46%) from Sub-Saharan Africa, 6 (11.54%) from Europe and Central Asia, 5 (9.62%) from Middle East and North Africa, 3 (5.77%) from South Asia, and 1 (1.92%) from Latin America and the Caribbean.

Considering Countries income level, 27 (51.92%) papers were from high income Countries, 12 (23.08%) were from upper-middle income Countries, 10 (19.23%) were from lower-middle income Countries and 3 (5.77%) were from low income Countries.^1^

Regarding the methodology of the studies, 32 (60.38%) were quantitative studies, 18 (33.96%) were qualitative studies, and 2 (3.77%) were mixed methods studies. Among qualitative studies, including those in mixed methods research, 15 (75.00%) were based on interviews, 1 (5.00%) on focus groups, 3 (15.00%) on both focus groups and interviews, and 1 (5.00%) on both interviews and analysis of responses to open ended questions in a survey. Among the quantitative studies, including those in mixed method research, 11 (32.35%) analyzed data from cohort studies, 22 (64.71%) were cross-sectional studies and 1 (2.94%) was a longitudinal study. As for the samples of qualitative studies, including those in mixed method research, the sample size ranged from 7 to 48 for focus groups, 6 to 67 for interviews and was of 79 participants for qualitative analysis of open-ended questions of a survey. For quantitative studies, including those in mixed method research, the sample size ranged from 259 to 8,009 for cohort studies, from 65 to 3,659 for cross-sectional studies and was of 92 women in the longitudinal study. Quantitative research was mainly based on ad hoc questionnaires or surveys, and data from medical records, thus making it more difficult to compare results.

The assessment of the quality of the included articles is shown in Table 2S. Evaluation using The Joanna Briggs Institute (Moola et al. 2020; https://jbi.global/critical-appraisal-tools) checklist found that all articles received an acceptable quality appraisal for inclusion in the current review. Of the 52 evaluated studies, 22 are cross-sectional studies: 13 articles had a low risk of bias score, 7 articles had a moderate risk of bias scores, and 2 articles had a high risk of bias scores. Out of the 12 assessed cohort studies, 7 articles had a low risk of bias score, 4 articles had a moderate risk of bias scores, and 1 article a high risk of bias scores. Out of the 18 assessed qualitative studies, 12 articles had a low risk of bias score and 6 articles had a moderate risk of bias score.

### Qualitative synthesis of results

Results were organized in 4 categories: (1) Work-life balance; (2) Women’s mental and physical health; (3) Job-related wellbeing and working experience; (4) Breastfeeding. Each paper could be included in one or more categories depending on its results.

#### Work-life balance

Six studies (11.54%) addressed work-life balance after returning from maternity leave. Many women who have returned to work reported that they would have liked a longer maternity leave (Juengst et al. 2019; Tohme and Abi-Habib 2022), and those employed in workplaces not supporting breastfeeding often use annual leave to stay longer with their children (Wolde et al. 2021). After returning to work, most women work the same or more hours per day than before their leave (Nguyen et al. 2022) and report tensions between work and care demands (Martins et al. 2019; Gregory 2021; Parcsi and Curtin 2013): women who continue breastfeeding after returning to work are those who experience more family-to-work conflict and overload (Spitzmueller et al. 2016). On the one hand, soon after returning to work, women emphasize the difficulty of accessing part-time positions, the lack of flexibility in the workplace, and the prevalence of a non-family-friendly occupational culture. On the other hand, in the long run, women are often forced to choose part-time precisely because of the difficulty in balancing career and private life (Gregory 2021).

#### Women’s mental and physical health

Three studies (5.77%) addressed women’s mental and physical health at work reentry. Women who experienced depression and anxiety almost every day during pregnancy report worse health while returning to work than those with no symptoms (Falletta et al. 2020). Moreover, taking a longer maternity leave is associated with women’s better physical and mental health at work reentry (Dagher et al. 2014a; Falletta et al. 2020). Finally, receiving coworkers’ and supervisors’ support during pregnancy is associated with lowest levels of prenatal stress, which are associated with lower incidence of postpartum depression after returning to work and quicker recovery times from birth-related injuries (Jones et al. 2022).

#### Job-related wellbeing and working experience

Five studies (9.62%) addressed job-related wellbeing and working experience at work re-entry. Nurses returning to work after the birth of their second child show a high level of work stress, with the highest score in their mother’s role commitment (Chen et al. 2022). Work stress seems higher in women with lower family monthly income, time since returning to work, and age of the first child, and in women with higher duration of maternity leave and higher depressive symptoms (Chen et al. 2022). On the other hand, women who have access to breastfeeding supporting conditions at their workplace report better job satisfaction (Wolde et al. 2021). Moreover, women express the need to feel valued for their work by their managers and coworkers, as this enables them to feel comfortable and confident with some of the compromises they made to balance work and family life (Parcsi and Curtin 2013).

As regards work productivity, it seems increased by working for pleasure, a healthy mother-infant bonding, and a positive experience of being back to work, and it seems positively correlated with maternal sense of competence (Tohme and Abi-Habib 2022). However, some women feel the need to try and compensate for their absence working harder than before, thereby showing that they are more committed to work than their colleagues (Oh and Mun 2022).

#### Returning to work and breastfeeding

Most of the studies identified by the systematic literature (*n* = 45, 86,54%) review addressed the impact of returning to work on breastfeeding duration and formula initiation.

Returning to work seems to constitute one of the most important barriers for exclusive breastfeeding or breastfeeding continuation (Bonet et al. 2013; Butudom et al. 2021; Dagher et al. 2016; Febrianingtyas et al. 2019; Gebrekidan et al. 2021; Gianni et al. 2019; Hendaus et al. 2018; Hmone et al. 2017; Horwood et al. 2018; Ickes et al. 2021a, b; Jain et al. 2022; Kobayashi and Usui 2017; Mabaso et al. 2020; Trafford et al. 2020; Wolde et al. 2021; Zhang et al. 2018): Kobayashi and Usui (2017) found that returning to work within a year after childbirth influence breastfeeding duration, but not initiation while de Lauzon-Guillain et al. (2019) found that returning to work influence both breastfeeding initiation and duration. Ahmadi and Moosavi (2013) found that 52.38% of employed mothers with infants aged 6–12 months use formula to feed their children, and 27.36% discontinued breastfeeding; while Jiravisitkul et al. (2022) found that 24% of employed mothers stopped breastfeeding within three months. Moreover, mothers report having early formula introduction or breastfeeding dismission specifically to prepare for their return to work (Hasan et al. 2020; Ickes et al. 2021a). In addition, some women report that they start worrying about their breastmilk stock right after returning to work, and that made them lose confidence with their ability to breastfeed (Febrianingtyas et al. 2019).

Research investigated several concurrent factors contributing to breastfeeding cessation and non-exclusive breastfeeding in working women. As regards to non-employment-related factors, motivation, satisfaction, and happiness while breastfeeding seems to promote breastfeeding continuation (Wolde et al. 2021). On the other hand, breastfeeding cessation and formula initiation seem to be connected to:

1. having quit smoking during pregnancy or having smoked before and during pregnancy (Castetbon et al. 2020);
2. having had a cesarean section (Castetbon et al. 2020);
3. being older (Xiang et al. 2016);
4. being single (Dagher et al. 2016);
5. having a higher education level (Bai et al. 2015; Dagher et al. 2016; Tsai 2013; Xiang et al. 2016), even if Horwood at al. (2018) found that higher education and being in the highest socio-economic tertile are risk factors for not practicing exclusive breastfeeding;
6. being supported by their partner and families (Gebrekidan et al. 2021; Riaz and Condon 2019; Tsai 2022);
7. having a better physical and mental health (Xiang et al. 2016).

Moreover, breastfeeding duration and exclusivity seem connected to breastfeeding habits and knowledge: breastfeeding cessation resulted lower in women with knowledge about breastfeeding (Gebrekidan et al. 2021; Sulaiman et al. 2018) and higher in women not pumping breast milk (Kebede et al. 2020) and with no family or friends who breastfed (Dagher et al. 2016). Lastly, the possibility to rely on parental childcare seem to promote breastfeeding continuation after returning to work (Bai et al. 2015).

For what concerns employment-related factors, a shorter duration of maternity leave appears to be related to the decision to stop breastfeeding or to start integrating formula (Ahmadi and Moosavi 2013; Aikawa et al. 2015; Bai et al. 2015; Kebede et al. 2020; Mandal et al. 2014; Mirkovic et al. 2014). In particular, Lubold (2016) found out that mothers returning to work within three months after giving birth breastfeed an average of five fewer weeks. Moreover, in primiparous women breastfeeding continuation was promoted by postponing return to work for at least 3 weeks after statutory postnatal maternity leave, while in women giving birth to either their first or second child postponing the return to work until at least 15 weeks was related to a higher prevalence of long breastfeeding duration (at least 6 months) (de Lauzon-Guillain et al. 2019).

Another factor that has been found to be particularly significant in several studies is the workload: women with shorter working hours (Bai et al. 2015; Tsai 2013) seem more likely to continue breastfeeding, while full-time employees (Kebede et al. 2020; Mirkovic et al. 2014; Lubold 2016), both at their first and second child (de Lauzon-Guillain et al. 2019), women working more than 6 h per day (Ahmadi and Moosavi 2013) or more than 20 h per week (Xiang et al. 2016; and women with excessive working hours (Jiravisitkul et al. 2022) maintain breastfeeding with more difficulty.

Breastfeeding seems to be hindered by many other aspects related to women’s profession and work organization. Managers (Xiang et al. 2016), professional workers (Dagher et al. 2016; Xiang et al. 2016), self-employed women (Castetbon et al. 2020; Xiang et al. 2016), women knowing the occupational legislation in force (Cervera-Gasch et al. 2020) and women working in workplaces offering childcare services (Jiravisitkul et al. 2022) seem more likely to continue breastfeeding after returning to work. On the other hand, breastfeeding cessation seems to be higher in:

1. intermediate employees and manual workers (Castetbon et al. 2020);
2. private organization employees (Kebede et al. 2020);
3. women working far from home (Jiravisitkul et al. 2022; Kebede et al. 2020);
4. women lacking flexible work time (Kebede et al. 2020; Riaz and Condon 2019);
5. women lacking childcare services (Riaz and Condon 2019).

For what concerns breastfeeding policies in the workplace, often women report discontinuing or reducing breastfeeding because of:

1. no breaks available for breastfeeding or pumping milk, especially for women working in the healthcare system (Ahmadi and Moosavi 2013; Jain et al. 2022; Jiravisitkul et al. 2022; Moulton et al. 2021; Riaz and Condon 2019);
2. no dedicated or at least suitable place for breastfeeding or pumping milk (Jain et al. 2022; Jiravisitkul et al. 2022; Soomro et al. 2017);
3. no suitable place to preserve expressed milk (Ahmadi and Moosavi 2013);
4. the lack of any workplace facilities that support breastfeeding apart from nursing breaks (Abou-Elwafa & El-Gilany 2019).

On the contrary, factors facilitating breastfeeding are:

1. breastfeeding support policies and accommodations (Cervera-Gasch et al. 2020; Wolde et al. 2021);
2. lactation room with dedicated space (Tsai 2013, 2022);
3. availability of breast pumping breaks (Tsai 2013, 2022);
4. participation in breastfeeding support groups (Cervera-Gasch et al. 2020);
5. childcare at the workplace (Jiravisitkul et al. 2022).

Such breastfeeding facilities seem to be slightly more provided in public and multinational companies (Soomro et al. 2017).

Another aspect that seems to play an important role in women’s choice of whether to continue breastfeeding once they return to work is perceived support in the workplace. The overall support to breastfeeding from their employers is considered insufficient to promote exclusive breastfeeding by many women (Desmond and Meaney 2016; Gebrekidan et al. 2021), who can experience discrimination and inappropriate comments connected to breastfeeding (Jain et al. 2022). Some workers feel so embarrassed by their breastfeeding status and perceive such a lack of support from their employers that even if they want to continue breastfeeding, they do not disclose to their employers that they are breastfeeding and do not make enquiries about being facilitated to continue to breastfeed, and that often lead to breastfeeding cessation (Desmond and Meaney 2016).

On the contrary, breastfeeding seems to be promoted by:

1. having a female supervisor (Cervera-Gasch et al. 2020);
2. positive colleagues’ attitude and coworker support (Jiravisitkul et al. 2022; Juengst et al. 2019), and in particular female coworker support (Zhuang et al. 2019).

Organizational cultures can also influence the perception of women’s own perceptions of breastfeeding in the workplace: some of them report their feeling that taking breast-pumping breaks can reduce their work efficiency and expressed feelings of guilt about taking time out to breastfeed (Burns and Triandafilidis 2019; Tsai 2022). Notably, only one study takes into account also job-related well-being, underlying that women who have access to supporting conditions at their workplace express not only better breastfeeding practice, but also better satisfaction with job (Wolde et al. 2021).

Finally, breastfeeding seems to be also connected to some characteristics of fathers’ work organization: the opportunity for fathers to work under a flextime system after childbirth increases both breastfeeding initiation and duration (Kobayashi and Usui 2017).

All the articles cited so far start from a perspective of promoting breastfeeding in the workplace, yet women who continue breastfeeding after returning to work experience more family-to-work conflict and overload than women who do not reconcile work and breastfeeding (Spitzmueller et al. 2016).

## Discussion

The World Health Organization (WHO) defines maternal mental health as “*a state of well-being in which a mother realizes her own abilities, can cope with the normal stresses of life, can work productively and fruitfully, and is able to make a contribution to her community*” (Herrman et al. 2005). Despite the WHO recommendations and the mounting evidence indicating high prevalence of maternal mental health impairment and its adverse impact on both mother and her infant, the maternal mental health agenda has not been incorporated into the primary health care system in most low- and middle-income countries (Atif et al. 2015).

Literature widely investigated mental health in women experiencing motherhood, with numerous studies focusing on the postpartum period and investigating prevalence and correlates of postpartum depression, psychosis, and the so-called “baby blues” (Bass III & Bauer, 2018; Degner 2017; Trigo 2021).

Mental health and job-related satisfaction of female workers with children are less studies, even if there is a growing literature on the topic in recent years (Cho and Jung 2022; Fleming and Kler 2014; Guler 2018).

With regard, specifically, to the experience of returning to work after maternity leave, the results of this systematic literature review show that this topic has received increasing attention in recent years, and it is certainly positive that alongside studies carried out in high income Countries and upper-middle income Countries, studies in lower-middle income Countries and low income Countries are also beginning to be counted. However, our results also show that the topic of re-entry at work remains understudied from the perspective of both clinical psychology and occupational psychology.

This represents an important gap in the literature, since if it is now well established that the postpartum experience entails a high burden for the somato-psychic balance of mothers, and if reconciling work and motherhood represents a challenge that often becomes strenuous for working women (Addati 2015; Collins 2019; Harvey 2016), returning to work following maternity leave represents a particularly critical time, since women have to renegotiate their role both in their relationship with their child, within the family and couple, and within the work context. It is precisely during this critical period that challenges and barriers may arise, and the way they are addressed and resolved, either positively or negatively, can impact on the quality of women’s personal and professional experience in the immediate future and years to come (Aitken et al. 2015; McCardel et al. 2022; Muzik et al. 2015; Saxbe et al. 2018).

It is striking this paucity of studies, since it is an experience that characterizes most working women who choose to have children: although there are still major disparities between female and male employment (Dashper 2019; Platt et al. 2016; Stamarski and Son Hing 2015), more and more women work (Levine and D’Agostino 2018; Rabinowitz and Rabinowitz 2021; Toossi and Morisi 2017) and the average age of first pregnancy in the world is 28 years (World Population Review 2023), an age within which a woman who can, wants and is allowed to work is likely to be effectively employed. Especially since women often wait until they have more stable employment to think about pregnancy (Lundborg et al. 2017; Seibert et al. 2016).

Another particularly significant aspect that emerges from our systematic review is that most studies exploring re-entry at work focus specifically on its impact on breastfeeding choices.

The studies considered emphasize how returning to work is often a barrier for exclusive breastfeeding or breastfeeding continuation (see among others Castetbon et al. 2020; Dagher et al. 2016; Ickes et al. 2021a; Riaz and Condon 2019; Sulaiman et al. 2018). A shorter duration of maternity leave, a higher workload and the lack of occupational policies supporting breastfeeding (such as breaks and places for breastfeeding or milk pumping), seem to be hindering factors (see among others Ahmadi and Moosavi 2013; Aikawa et al. 2015; Bai et al. 2015; Jain et al. 2022; Jiravisitkul et al. 2022; Kebede et al. 2020; Lubold 2016; Mirkovic et al. 2014; Xiang et al. 2016). On the other hand, partner and family support, a better physical and mental health, and the opportunity for fathers to work under a flextime system after childbirth seem to increase both breastfeeding initiation and duration (Gebrekidan et al. 2021; Kobayashi and Usui 2017; Riaz and Condon 2019; Tsai 2022; Xiang et al. 2016).

Considering the number of studies on breastfeeding, it is alarming the extreme paucity of research on women’s lived experience. This is even more unfortunate since the few articles with a wider perspective on breastfeeding suggest important lines of research that deserve further investigation. Indeed, one study explored satisfaction and happiness while breastfeeding, underlying their role in promoting breastfeeding continuation (Wolde et al. 2021), while another study report that women who continue breastfeeding after returning to work seem to experience more family-to-work conflict and overload (Spitzmueller et al. 2016). There are no studies deepening such factors, as well as no study considered the experience of those women for whom choosing to discontinue breastfeeding represented a syntonic and functional choice at that point in their motherhood journey. Putting women who would like to continue breastfeeding in a position to do so does not imply silencing the experience of those women who do not wish to do so or have felt liberated from the burden of having to do so.

Broadening the scope from just breastfeeding, there are also few studies on the family-work balance experienced upon re-entry at work, women’s satisfaction in the workplace, and how their way of working changes after maternity leave. The studies that are there emphasize the struggle experienced to conciliate work and care demands (Martins et al. 2019; Gregory 2021; Parcsi and Curtin 2013). Even if work productivity and maternal sense of competence do not seem to be in conflict (Tohme and Abi-Habib 2022), women often feel the need to try and compensate for their absence working harder than before (Oh and Mun 2022), as if maternity leave and motherhood were something to be ashamed of. Finally, women’s physical and mental health, a widely studied topic in the postpartum period, is also poorly investigated when focusing on return to work (Dagher et al. 2014b; Falletta et al. 2020; Jones et al. 2022).

These are issues that should be further investigated. It is not enough to investigate mental health and job satisfaction in women returning to work after maternity leave: research should also explore the connections between these aspects and what motivated the woman’s choice to work first and return to work later; workplace conditions, policies and cultures; how being workers and mothers is experienced in the work context, in the couple and in the family; how the woman experienced pregnancy and childbirth; how she experienced and continues to experience the relationship with her child and the balance between family and career; and so on. Moreover, there is a need for studies that explore the relationship between all this facets and parental leave policies, paternity leave policies, if and how the couple shares household and parenting, as well as social and family expectations in this regard. The possible lines of research are broad and just waiting to be wrought.

## Conclusions

The results of this systematic review of recent research on women’s experience of returning to work following maternity leave allows for a deeper look at the topic of work-life balance both by looking at the results that have been found and by looking at the aspects that are lacking.

Indeed, it is surprising that such a sensitive aspect of a growing proportion of the population is largely under-studied, except as regards its impact on breastfeeding choices. There is a particular lack in longitudinal studies tracking the mental and occupational well-being of working women in the trajectory leading from the desire for motherhood, to pregnancy, to return to work. Such studies would allow for an in-depth study of the relationship between work, motherhood, mental health, and work-related satisfaction, and their relationship with type of occupation, characteristics of work organization, type of family structure, organization of family and couple life, geographical areas of origin, socioeconomic status, and so on.

On the other hand, it is necessary to promote a cultural change even within the scientific landscape that allows to reverse the proportion of studies proportion of studies exploring returning to work after maternity leave focusing on the impact on breastfeeding to studies focusing on how women experience this transition, considering their mental and physical health, job satisfaction, family experience and relationship with their child. This would make it possible to avoid reproducing, even at the academic level, an attitude that focuses on only one aspect of women’s identity, namely motherhood, and on specific functions of this identity, namely breastfeeding, instead of taking up the challenge of constructing a prismatic image of women’s identity that encompasses being a worker, partner, mother, daughter, etc. A person, in its different declinations.

The present study has several limitations that must be considered in generalizing the results. First, the samples of the studies included in the review are very heterogeneous and come from different socio-cultural backgrounds. Moreover, the heterogeneity of the research methods adopted and of the instruments employed in the reviewed studies (mainly ad hoc questionnaires or surveys for quantitative studies) may have affected the results. Finally, some important results may have been missed by this review because only publications in English were considered, thus potentially overlooking studies that could corroborate or disconfirm the reported data.

## Electronic supplementary material

Below is the link to the electronic supplementary material.

Supplementary Material 1

## Footnotes

1 Data based on the World Bank current Countries classification by income.
