# Supplementary material for: Returning to work after maternity leave: a systematic literature review
Source: Arch Womens Ment Health. 2024 Apr 5;27(5):737–49. doi: 10.1007/s00737-024-01464-y (PMC11405436; doi:10.1007/s00737-024-01464-y)
Supplement: Supplementary file 1 — Supplementary Material 1 [file 737_2024_1464_MOESM1_ESM.docx]

**SUPPLEMENTARY MATERIAL**

**Table 1. Results of the systematic review on returning to work after maternity leave**

| **ID** | **Year of publication** | **Authors** | **Title** | **Journal** | **Study methodology** | **Sample** | **Main findings** | **Measures** | **Country** |
| --- | --- | --- | --- | --- | --- | --- | --- | --- | --- |
| 1 | 2022 | Chen K.; Wei L.; Zhang Y.; Jiang W.; Wang J.; Pan Y. | Work stress in nurses returning to tertiary a general hospitals in China after the delivery of their second child: a cross-sectional study | BMC Health Services Research | Cross-sectional study | 448 nurses returning to work after the birth of their second child, working in 23 general hospitals in China. | The total work stress score of returning nurses after giving birth to their second child was high (90.40 ± 18.29) and the dimension with the highest score was the “mother’s role commitment”. Work stress seems higher in women with lower family monthly income, time since returning to work, and age of the first child, and in women with higher duration of maternity leave and higher depressive symptoms. | 1. Ad hoc general information questionnaire; 2. postnatal return-to-work stress scale; 3. Self-rating Depression Scale (SDS) | China |
| 2 | 2022 | Jain S, Neaves S, Royston A, Huang I, Juengst SB. | Breastmilk pumping experiences of physician mothers: quantitative and qualitative findings from a nationwide survey study | J Gen Intern Med | Cross-sectional study | 724 physician mothers (120 trainees vs 604 attending. 90.8% trainees had only 1 child, attendees were more likely to have ≥2 children. | 40% trainees reported difficulties accessing adequate facilities or time for pumping (vs 28% attendees), similar percent breastfeeding or pumping breast milk at work (55% of trainees vs 45% of attendees). Needing to be present for clinical training as a time barrier: trainees (56.2%) vs attendees (38.0%). Attendees (50.6%) reported inconvenience to patients being a more common barrier than trainees (32.9%). Experiencing discrimination: trainees (34.5%) vs attendees (23.7%). Inappropriate comments: trainees (41.4%) vs attendees (29.1%). Areas deserving the highest attention (themes): lack of support from leaders; inadequate time and unsanitary, inconvenient, and crowded lactation spaces; barriers of time, support, and space; absent or sub-optimal legal and financial accommodations. | Ad hoc survey | USA |
| 3 | 2022 | Jiravisitkul P.; Thonginnetra S.; Kasemlawan N.; Suntharayuth T. | Supporting factors and structural barriers in the continuity of breastfeeding in the hospital workplace | International Breastfeeding Journal | Mixed-methods study: cross sectional study + qualitative study | Quantitative study: 65 mothers, doctor or dentist (25%), nurse (25%), nursing assistant (18%), and others (32%). Average duration of maternity leave = 95 days. Mean age 33.98 (4.11). Qualitative study: 7 mothers, divided into subgroups of successful (n = 4) and unsuccessful (n = 3) continuous breastfeeding after a return to work. | 90% participants intended to EBF for six months and made prenatal breastfeeding preparations. 77% were continuing to breastfeed when returning to work, 24% (12/50) stopped breastfeeding within three months. Main reasons for discontinuing breastfeeding at work: no break for pumping milk (27/50, 54%), no place to express milk (25/50, 50%), excessive working hours (13/50, 26%), distance between home and the workplace (9/50, 18%). 24% (12/50) reported no barriers at work. 23/59 wanted to send infant to childcare, 12/23 were able to do so. Supporting factors for sustaining breastfeeding: childcare at the workplace and colleagues' attitude. | Quantitative study: ad hoc questionnaire; qualitative study: focus groups were conducted | Thailand |
| 4 | 2022 | Jones K.P.; Brady J.M.; Lindsey A.P.; Cortina L.M.; Major C.K. | The Interactive Effects of Coworker and Supervisor Support on Prenatal Stress and Postpartum Health: A Time-Lagged Investigation | Journal of Business and Psychology | Longitudinal study | 92 mothers who completed the follow-up survey, from a sample of 118 pregnant employees, average age 31.9 years, from 4 to 38 weeks pregnant (M = 22.5 weeks), 70% pregnant with their first child. | Support of coworkers and supervisors during pregnancy was associated with lowest levels of prenatal stress, which were associated with lower incidence of postpartum depression and quicker recovery times from birth-related injuries. Higher levels of stress during pregnancy associated with increased incidence of postpartum depression. Significant indirect effects and aspects of workplace environment: when perceptions of supervisor support were higher, coworker support during pregnancy predicted lower incidence of postpartum depression and quicker recovery times through reduced prenatal stress. | Ad hoc web-based surveys | USA |
| 5 | 2022 | Nguyen T.T.; Cashin J.; Tran H.T.T.; Vu D.H.; Nandi A.; Phan M.T.; Van N.D.C.; Weissman A.; Pham T.N.; Nguyen B.V.; Mathisen R. | Awareness, Perceptions, Gaps, and Uptake of Maternity Protection among Formally Employed Women in Vietnam | International Journal of Environmental Research and Public Health | Mixed-methods study: cross sectional study + qualitative study | Survey: 494 women working in the formal sector, 107 pregnant women and 387 mothers of infants. Interviews: 39 women, 9 pregnant, 30 mothers of infants. | Of 182 mothers with infants aged 6–11 months: 30 (16.5%) returned to work before accruing 180 days of maternity leave. Of 121 women who had returned to work: 26 (21.5%) did not receive a one-hour paid break every day to express breastmilk, relax, or breastfeed, 46 (38.0%) worked the same or more hours per day than before maternity leave. | Quantitative study: ad hoc survey; qualitative study: interviews | Vietnam |
| 6 | 2022 | Oh E.; Mun E. | Compensatory Work Devotion: How a Culture of Overwork Shapes Women’s Parental Leave in South Korea | Gender and Society | Qualitative study | 64 college-educated women, median age 35, average number of children 1.3. | After taking leave, "compensatory work devotion": women try to compensate for their absence by working harder than before, thereby showing that they are more committed than their colleagues. | In-depth interviews | South Korea |
| 7 | 2022 | Tohme P, Abi-Habib R. | Correlates of work productivity and maternal competence after having a baby: the roles of mother-infant bonding and maternal subjective experiences | BMC Womens Health | Cross-sectional study | 97 mothers, age 25-42 years (M = 32.52, SD = 3.61), with children 6-24 months of age. 97% employed. | Maternity leave duration between 7 and 365 days (M = 75.70. SD = 40.81), 91% wanted to have had longer maternity leave. 86 breastfed their child. Significant correlations between work productivity and maternal sense of competence, irrespective of the length of maternity leave. Significant predictors of work productivity: working for pleasure, mother-infant healthy bonding, positive subjective experience of being back to work. Maternal stress predicted maternal competence. | 1. Health and Work Questionnaire (HWQ); 2. ad hoc questions; 3. Parenting Sense of Competence (PSOC); 4. Postpartum Bonding Questionnaire (PBQ); 5. Parental Stress Scale (PSS); 6. Guilt about Parenting Scale (GAPS); 7Short Experiences in Close Relationships-Revised (ECR-R short) | Lebanon |
| 8 | 2022 | Tsai S.-Y. | Shift-work and breastfeeding for women returning to work in a manufacturing workplace in Taiwan | International Breastfeeding Journal | Cross-sectional study | 715 employed mothers, with either shift work (n = 334, 99 age 20-29 years, 235 ≥ 30 years), or non-shift work (n = 381, 72 age 20-29 years, 309 age ≥ 30 years). | Encouragement by peers to continue lactation after returning to work was not significantly different between shift and non-shift workers. Feeling that taking two breast-pumping breaks can reduce a mother’s work efficiency: shift workers vs non-shift workers (56.28 vs. 49.60%; p < 0.01). Shift workers: 90.1% breastfed during maternity leave, rates after returning to work decreased to 21.5% (1-6 months) and 17.9% (more than 6 months). Non-shift workers: 87.6% breastfed during maternity leave, rates after returning to work were 24.1% (1-6 months) and 34.6% (more than 6 months). Significant factors for continuing to breastfeed: using a lactation room and taking advantage of breast-pumping breaks. Among non-shift workers, positive effects partner support (OR = 4.89) toward a mother continuing breastfeeding for more than six months after returning to work. | Ad hoc questionnaires | Taiwan |
| 9 | 2021 | Gebrekidan K.; Plummer V.; Fooladi E.; Hall H. | Attitudes and experiences of employed women when combining exclusive breastfeeding and work: A qualitative study among office workers in Northern Ethiopia | Maternal and Child Nutrition | Qualitative study | 20 mothers working full-time outside home, with babies of 12 months or younger. | 3 themes emerged (mothers' knowledge, attitudes, and practice towards breastfeeding; workplace context and employment conditions; support received at home) as contributing factors to continuing exclusive breastfeeding. Participants reported that the overall support given to breastfeeding women from their employers was insufficient to promote exclusive breastfeeding. | Interviews | Ethiopia |
| 10 | 2021 | Gregory S.K. | Managing labour market re-entry following maternity leave among women in the Australian higher education sector | Journal of Sociology | Qualitative study | 15 female higher education employees aged 27-43 years at first interview. 9 pregnant at the first interview, 6 recently had a child in the last 12 months to 3 years, 8 employed full time. | Women reported tensions between their paid work/care demands; gender inequality, challenges of returning to employment; lack of access to part-time position, flexibility and/or family-friendly workplace culture. By the third interview, there were only two full-time employees, compared with eight at interview one (part-time as a ‘constrained choice’). | Interviews | Australia |
| 11 | 2021 | Ickes S.B.; Oddo V.M.; Sanders H.K.; Nduati R.; Denno D.M.; Myhre J.A.; Kinyua J.; Iannotti L.L.; Singa B.; Farquhar C.; Walson J.L. | Formal maternal employment is associated with lower odds of exclusive breastfeeding by 14 weeks postpartum: A cross-sectional survey in Naivasha, Kenya | American Journal of Clinical Nutrition | Cross-sectional study | 1,186 mothers (n = 296 at hospital discharge, n = 298 at 6 weeks, n = 295 at 14 weeks, and n = 297 at 36 weeks). Mean age 27.2 years. Mean ± SE maternity leave lengths = 13.0 ± 1.0 and 13.2 ± 1.2 weeks among formally employed and commercial farmworkers. | Reason for discontinuing EBF: returning to work (46.5%), belief that it is appropriate to introduce other foods based on the child’s age (33.5%) and perceived milk insufficiency (13.7%). Mothers of 14-wk-old children were more likely to report returning to work as the primary reason for EBF cessation than mothers surveyed at 9 months (60.6% ± 0.05 compared with 38.9% ± 0.03, respectively; P < 0.001). | Ad hoc survey | Kenya |
| 12 | 2021 | Ickes S.B.; Sanders H.; Denno D.M.; Myhre J.A.; Kinyua J.; Singa B.; Lemein H.S.; Iannotti L.L.; Farquhar C.; Walson J.L.; Nduati R. | Exclusive breastfeeding among working mothers in Kenya: Perspectives from women, families and employers | Maternal and Child Nutrition | Qualitative study | 42 mothers employed in a flower farm or hotel. Mothers' mean age 29.7 (1.1) years. Mean (SE) age of their youngest child = 4.5 (0.6) months. Among mothers with a child under 6 months (n = 21), one (5%) had stopped breastfeeding, n = 13 (62%) were currently practicing mixed feeding and 7 (33%) reported exclusively breastfeeding. | Early cessation of EBF in preparation for return to work. Managers reported supporting mothers through flexible work hours and duties. Challenges when returning to work: lack of proximate or on-site childcare, low support for and experience with milk expression. | Semi-structured, in-depth, key informant interviews | Kenya |
| 13 | 2021 | Moulton K.L.; Battaglioli N.; Sebok-Syer S.S. | Is Lactating in the Emergency Department a Letdown? Exploring Barriers and Supports to Workplace Lactation in Emergency Medicine | Annals of Emergency Medicine | Qualitative study | 24 mothers working in emergency departments: physicians, fellows, and research faculty (n = 17), medical students and residents (n = 4), and nurses (n = 3). At least 1 return-to-work experience after giving birth within the prior 3 years. | 3 themes: (1) emergency medicine culture, (2) workplace lactation policies, (3) supports for workplace lactation. Cultural barriers to desired lactation habits, despite formalized workplace lactation policies. | Semi-structured individual interviews | USA |
| 14 | 2021 | Wolde FB, Ali JH, Mengistu YG. | Employed mothers' breastfeeding: Exploring breastfeeding experience of employed mothers in different work environments in Ethiopia | PLoS One | Qualitative study | 17 full-time women employees who have a currently breastfeeding child of two years or younger, mean age 30 years. | Returning to work at three months was the major barrier to continuous breastfeeding. Mothers who have access to supporting conditions at their workplace expressed better breastfeeding practice and better satisfaction with job. (Theme 1) workplace barriers and facilitators to breastfeeding: mothers’ reported motivation, satisfaction, and happiness while breastfeeding, physical and emotional challenges to continue breastfeeding after returning to work, difference in health status of breastfed and non-breastfed child. (Theme 2) addressing barriers: mothers with no supporting condition used annual leave to stay longer with child; they used spare time at work to breastfeed. | Interviews (semi-structured questions + open ended questions) | Ethiopia |
| 15 | 2021 | Butudom A.; McFarlin B.L.; Klima C.S.; Spatz D.L.; Kennelly J.F.; McCreary L.L.; Patil C.L.; Koenig M.D. | Behavior outcomes of breastfeeding-friendly policies among thai mothers working in a factory: A descriptive study | Pacific Rim International Journal of Nursing Research | Cross-sectional study | 216 mothers. Years of work in the factory: 51.9% between 5-9. 70.8% between 25-34 years of age. | 12% discontinued pumping and breastfeeding because of returning to work. Factory’s levels of breastfeeding support: 26.1% excellent, 59% very good. | 1. Ad hoc demographic questionnaire; 2. Breastfeeding in the Workplace Survey Questionnaire (BWSQ) | Thailand |
| 16 | 2020 | Castetbon K, Boudet-Berquier J, Salanave B. | Combining breastfeeding and work: findings from the Epifane population-based birth cohort | BMC Pregnancy Childbirth | Cohort study | 1,487 women who had worked before pregnancy and returned to work within a year after delivery (from a total sample of 2,480 women). Median time of returning to work: 5.3 months. | Percentages of returning to work within one year: 85.1% women who did not breastfeed at all, 83.0% women who breastfed for less than 1 month, 85.3% women who breastfed for 1–4 months, 77.1% those who breastfed for more than 4 months (p = 0.0002). Only women who breastfed for more than 4 months returned to work much later. 34.5% combined breastfeeding and work and breastfed for a longer duration. 93.7% of women who continued to breastfeed after returning to work had exclusively breastfed at birth, vs 75.2% of those who ceased to breastfeed before returning to work. | Ad hoc questions administered in person, by phone and online, records from medical files | France |
| 17 | 2020 | Cervera-Gasch Á.; Mena-Tudela D.; Leon-Larios F.; Felip-Galvan N.; Rochdi-Lahniche S.; Andreu-Pejó L.; González-Chordá V.M. | Female employees’ perception of breastfeeding support in the workplace, public universities in Spain: A multicentric comparative study | International Journal of Environmental Research and Public Health | Cross-sectional study | 301 women from two universities, mean age 41.94 years (SD = 4.55, Min = 31, Max = 53; p = 0.333). 162 teachers/researchers and 139 administration/services staff. | Intention to continue breastfeeding when back at work reported by 70.4%, 57.8% continued breastfeeding after returning to work. Factors associated with continuing breastfeeding: university having a breastfeeding support policy and special accommodation (p < 0.001); participating in breastfeeding support groups (p < 0.001); intending to continue breastfeeding after returning to work (p < 0.001); knowing the occupational legislation in force (p = 0.009); having a female supervisor (p = 0.04). | 1. Ad hoc sociodemographic questionnaire; 2. Workplace Breastfeeding Support Scale (WBSS) | Spain |
| 18 | 2020 | Falletta L.; Abbruzzese S.; Fischbein R.; Shura R.; Eng A.; Alemagno S. | Work Reentry After Childbirth: Predictors of Self-Rated Health in Month One Among a Sample of University Faculty and Staff | Safety and Health at Work | Cross-sectional study | 249 women who had given birth in the past 5 years. Mean age 34. Most women took 12 or fewer weeks off for maternity leave. | Women who experienced depression (odds ratio [OR] = 0.096 [95% confidence interval {CI} = 0.019 to 0.483, p = 0.004]) and anxiety (OR = 0.164, [95% CI = 0.042 to 0.635, p = 0.009]) nearly every day reported worse health at work reentry than those with no symptoms. Taking a longer maternity leave (OR = 14.552 [95% CI = 4.934 to 42.918, p < 0.001]) was associated with reporting better health at work reentry. | Ad hoc questionnaires | USA |
| 19 | 2020 | Hasan A.M.R.; Smith G.; Selim M.A.; Akter S.; Khan N.U.Z.; Sharmin T.; Rasheed S. | Work and breast milk feeding: a qualitative exploration of the experience of lactating mothers working in ready made garments factories in urban Bangladesh | International Breastfeeding Journal | Qualitative study | Focus groups: 19 mothers. Interviews: 8 mothers. | Main themes: knowledge and experience of breastfeeding; structural barriers (home and workplace); consequences of inadequate breastfeeding; perception and experience of using expressed breast milk. Most mothers introduced formula as early as 2 months to prepare for return to work. Barriers impeding EBF: excessive workload, inadequate crèche facilities at work, lack of adequate caregivers at home. Very little knowledge of use of expressed breast milk. | Interviews (in-depth and key informant) + focus group discussions | Bangladesh |
| 20 | 2020 | Kebede T, Woldemichael K, Jarso H, Bekele BB. | Exclusive breastfeeding cessation and associated factors among employed mothers in Dukem town, Central Ethiopia | Int Breastfeed J | Cross-sectional study | 313 employed mothers (6 to 24 months children) working in governmental and nongovernmental organizations, mean age 27.1 (SD = 3.44) years. | Prevalence of exclusive breastfeeding cessation 75.7% (95% CI 71.0, 80.5%). Cessation of EBF associated factors: short duration of maternity leave (AOR 9.3; 95% CI 3.8, 23), full time employee (AOR 3.5; 95% CI 1.7, 11), private organization employee (AOR=2.1, 95% CI(1, 4.3)), lack of flexible work time (AOR 3.0; 95% CI 1.2, 7.5), not pumping breast milk (AOR 4.3; 95% CI 1.7, 11), lack of a lactation break (AOR 6.7; 95% CI 3,14.5), work place far away from child (AOR 3.1; 95% CI 3.1, 6.3). | Ad hoc survey | Ethiopia |
| 21 | 2020 | Mabaso B.P.; Jaga A.; Doherty T. | Experiences of workplace breastfeeding in a provincial government setting: a qualitative exploratory study among managers and mothers in South Africa | International Breastfeeding Journal | Qualitative study | 8 mothers. | 3 critical maternity periods: pregnancy, maternity leave, and return to work. Lack of knowledge about comprehensive maternity benefits. Most participants stopped breastfeeding prior to or immediately upon return to work. Barriers to breastfeeding continuation: absence of conversation about infant feeding plans between managers and mothers. | Semi-structured interviews | South Africa |
| 22 | 2020 | Trafford Z.; Jewett S.; Swartz A.; Lefevre A.E.; Winch P.J.; Colvin C.J.; Barron P.; Bamford L. | Reported infant feeding practices and contextual influences on breastfeeding: qualitative interviews with women registered to MomConnect in three South African provinces | International Breastfeeding Journal | Qualitative study | 115 women registered to MomConnec: 67 involved in individual interviews and 48 in focus group discussions | Returning to work or school sometimes prevented 6 months of EBF. | Individual in-depth interviews + focus group discussions | South Africa |
| 23 | 2019 | Abou-Elwafa H.S.; El-Gilany A.-H. | Maternal work and exclusive breastfeeding in Mansoura, Egypt | Family Practice | Cross-sectional study | 633 working mothers attending health care facilities for vaccinating their infants aged 6 months | Exclusive breastfeeding rate was 14.1%. Results underline that EBF rate is low among working mothers return to work after 4 months by delivery. | Ad hoc questionnaires | Egypt |
| 24 | 2019 | Burns E, Triandafilidis Z. | Taking the path of least resistance: a qualitative analysis of return to work or study while breastfeeding | Int Breastfeed J | Qualitative study (we included only the results of the qualitative study of a mixed method research) | Interviews: 10 mothers employed as staff (n = 8) or enrolled as students (n = 2) at the university. Survey: 79 mothers, most of them were born in Australia [66%], had spent one to 5 years at the university [53%], were no longer breastfeeding [63%], and had one [49%] or two [28%] children. | The analysis revealed four themes. The first theme explores staff and students’ experiences of maternity leave, flexible work arrangements, campus childcare, and their relationships with supervisor and colleagues. The second theme experiences of using designated spaces suitable to breastfeed and express breast milk, and their experiences related to storage of breast milk. The third theme reflects women’s experiences of mixing professional and personal lives.  Feelings of guilt about taking time out to breastfeed were a feature of women’s experiences of returning to work at university. Returned to work mums identified that by the time they returned to work they would only need to feed before and after work and could manage the daytime by expressing for comfort if needed. | In-depth interviews + open ended questions from a survey | Australia |
| 25 | 2019 | Dal Forno Martins G.; Leal C.L.; Schmidt B.; Piccinini C.A. | Motherhood and work: Experience of women with established careers | Trends in Psychology | Qualitative study | 3 public employees mothers. | Since pregnancy, concerns regarding changes and reconciliation of maternal and professional demands arises. Feelings of insecurity and ambivalence were also present when babies entered daycare center and women returned to work. A sense of overload by the activities after the baby’s entrance in daycare center and the mother’s return to work were evidenced. | Interviews | Brazil |
| 26 | 2019 | de Lauzon-Guillain B, Thierry X, Bois C, Bournez M, Davisse-Paturet C, Dufourg MN, Kersuzan C, Ksiazek E, Nicklaus S, Vicaire H, Wagner S, Lioret S, Charles MA. | Maternity or parental leave and breastfeeding duration: Results from the ELFE cohort | Matern Child Nutr | Cohort study | 8,009 mother-child pairs. | Older infant age at maternal return to work was related to greater likelihood to initiate breastfeeding and longer breastfeeding duration among breastfeeding mothers. Among primiparous women, both postponing return to work for at least 3 weeks after statutory postnatal maternity leave and working less than full‐time at 1-year post‐partum (as compared with full‐time) were related to higher prevalence of breastfeeding initiation. Among women giving birth to their first or second child, postponing the return to work until at least 15 weeks was related toa higher prevalence of long breastfeeding duration (at least 6 months) as compared with intermediate duration (3 to <6 months). Working part‐time was also positively related to breastfeeding duration. | Ad hoc questions administered in person and by phone, records from medical files. Data were collected from the ELFE (Etude Longitudinale Française depuis l'Enfance) study | France |
| 27 | 2019 | Febrianingtyas Y.; Februhartanty J.; Hadihardjono D.N. | Workplace support and exclusive breastfeeding practice: A qualitative study in Jakarta, Indonesia | Malaysian Journal of Nutrition | Qualitative study | 18 working mothers. | Mothers revealed that upon returning to work, their breastfeeding performance was reduced because of the need to adapt their breastfeeding/breastmilk expression to the home and office. Moreover, mothers were worried about their breast milk stock right after returning to work, and that made them lose confidence with their ability to breastfeed. | Interviews | Indonesia |
| 28 | 2019 | Gianni M.L.; Bettinelli M.E.; Manfra P.; Sorrentino G.; Bezze E.; Plevani L.; Cavallaro G.; Raffaeli G.; Crippa B.L.; Colombo L.; Morniroli D.; Liotto N.; Roggero P.; Villamor E.; Marchisio P.; Mosca F. | Breastfeeding difficulties and risk for early breastfeeding cessation | Nutrients | Cross-sectional study | 552 mothers | Return to work (OR=7.65; p= <0.0001) was associated with a higher risk of non-exclusive breastfeeding at three months of delivery. | Ad hoc questionnaires | Italy |
| 29 | 2019 | Juengst S.B.; Royston A.; Huang I.; Wright B. | Family Leave and Return-to-Work Experiences of Physician Mothers | JAMA Network Open | Cross-sectional study | 844 physician mothers aged between 27-67 years | Of the women surveyed, 619 (73.3%) felt that leave time was insufficient. The majority (751 [89.0%]) would have preferred 11 weeks to 6 months of leave vs the 5 to 12 weeks (often not paid) most commonly available. The most frequently reported negative experiences when returning to work were associated with lack of facilities for breast pumping (range, 12 of 78 [15.4%] for the third child to 272 of 844 [32.2%] for the first child) and time for breast pumping (range, 27 of 78 [34.6%] for the third child to 407 of 844[48.2%] for the first child), difficulty obtaining childcare (e.g., for the first child, 298 of 844 [35.3%]),and discrimination (eg, for the first child, 152 of 844 [18.0%]).  The most common positive experience was emotional support (e.g., for the first child, 504 of 844 [59.7%]), primarily from colleagues. | Ad hoc survey | USA |
| 30 | 2019 | Riaz S, Condon L. | The experiences of breastfeeding mothers returning to work as hospital nurses in Pakistan: A qualitative study | Women Birth | Qualitative study | 7 mothers returning to full-time work as nurses in a tertiary hospital. | Themes identified were: a child’s right to breastfeed, institutional power and family support to maintain breastfeeding. Returning to work mothers encountered rigid hospital policies and practices, such as a short and non-negotiable period of maternity leave, inflexible shift patterns, and lack of childcare services | Semi-structured interviews | Pakistan |
| 31 | 2019 | Zhuang J.; Bresnahan M.J.; Yan X.; Zhu Y.; Goldbort J.; Bogdan-Lovis E. | Keep Doing the Good Work: Impact of Coworker and Community Support on Continuation of Breastfeeding | Health Communication | Cross-sectional study | 500 working mothers aged between 18 and 35 years | Perception of coworker support was the only significant predictor of mothers’ decision to continue breastfeeding after returning to work, β = .50, t = 9.44, p < .001. | Ad hoc surveys, with sone items adapted from existing research | USA |
| 32 | 2018 | Hendaus M.A.; Alhammadi A.H.; Khan S.; Osman S.; Hamad A. | Breastfeeding rates and barriers: A report from the state of Qatar | International Journal of Women's Health | Cross-sectional study | 453 mothers | Among the most common barriers to breastfeeding, 16.3% women reported returning to work. | Ad hoc telephone survey | Qatar |
| 33 | 2018 | Horwood C.; Haskins L.; Engebretsen I.; Phakathi S.; Connolly C.; Coutsoudis A.; Spies L. | Improved rates of exclusive breastfeeding at 14 weeks of age in KwaZulu Natal, South Africa: What are the challenges now? | BMC Public Health | Cross-sectional study | 3,659 mothers (from a sample of 4,172 caregivers) of 14-week-old infants. Among mothers 49.8% were exclusive breastfeeding, 23.1% were mixed breastfeeding and 27.0% were not breastfeeding. Among non-maternal caregivers 11.8% reported exclusive breast feeding, 23.4% mixed breastfeeding and 62.3% were not giving breastmilk. | Higher education (OR 0.6, 95% CI 0.4–0.8) and being in the highest socio-economic tertile (OR 0.7, 95% CI 0.6–0.9) are risk factors for not practicing exclusive breastfeeding. However, returning to work (OR 0.3, 95% CI0.2–0.3) and school (OR 0.2 95% CI, 0.1–0.3) were most strongly associated with less exclusive breastfeeding. | Ad hoc survey | South Africa |
| 34 | 2018 | Sulaiman Z, Liamputtong P, Amir LH. | Timing of return to work and women's breastfeeding practices in urban Malaysia: A qualitative study | Health Soc Care Community | Qualitative study | 40 working women with a mean age of 32 years. | Most women (75%) returned to work between 2 and 3 months. Only 10% returned to work 4 months or later postpartum, and 15% had an early return to work (defined here as less than 2 months). Women fell into three groups: Passionate women with a strong determination to breastfeed, who exclusively breastfed for 6 months; Ambivalent women, who commenced breastfeeding but were unable to sustain this after returning to work; and Equivalent women, who perceived formula feedings equally nutritious as breast milk. Although longer maternity leave was very important for Ambivalent women to maintain breastfeeding, it was not as important for the Equivalent or Passionate women. In conclusion, returning earlier was not an absolute barrier to continuing breastfeeding. Instead, a woman’s beliefs and perceptions of breastfeeding were more important than the timing of her return to work in determining her ability to maintain breastfeeding or breast milk feeding. | Interviews | Malaysia |
| 35 | 2018 | Zhang Y.; Jin Y.; Vereijken C.; Stahl B.; Jiang H. | Breastfeeding experience, challenges and service demands among Chinese mothers: A qualitative study in two cities | Appetite | Qualitative study | In-depth interviews: 10 mothers in Shangai, 10 mothers in Weifang. Focus groups: 6 mothers in each focus group, 5 realized in Shangai and 5 in Weifang. | Returning to work constituted the most important barrier to continued breast feeding to six months for working mothers. | Individual in-depth interviews + focus group discussions | China |
| 36 | 2017 | Hmone M.P.; Li M.; Agho K.; Alam A.; Dibley M.J. | Factors associated with intention to exclusive breastfeed in central women's hospital, Yangon, Myanmar | International Breastfeeding Journal | Qualitative study (we included only the results of the qualitative study of a mixed method research) | 24 women. | Returning to work was the main barrier to continue breastfeeding | In-depth interviews | Myanmar |
| 37 | 2017 | Kobayashi M.; Usui E. | Breastfeeding practices and parental employment in Japan | Review of Economics of the Household | Cohort study | 3,651 parents and 7,148 children. | Compared to mothers who leave their jobs after childbirth, mothers who return to work within a year after childbirth do not significantly differ in breastfeeding initiation but have a breastfeeding duration which is shorter by 1.654 months. On the other hand, when fathers work under a flextime system after childbirth, breastfeeding initiation is higher, and the duration is longer by 4.418 months. | Surveys. Data were collected from the Japanese Longitudinal Survey on Employment and Fertility (LOSEF) | Japan |
| 38 | 2017 | Soomro JA, Shaikh ZN, Bijarani SA, Saheer TB. | Factors affecting breastfeeding practices among working women in Pakistan | East Mediterr Health J | Cross-sectional study | 297 mothers. | Mothers from 36 (12.1%) sites reported receiving breastfeeding breaks, and 86% of the mothers had received 3 months paid maternity leave. Provision of a lighter job and information about breastfeeding options on return to work were reported from 15% and 5% of the workplaces, respectively. Only two sites had designated breastfeeding corners. Significantly different results were found between types of employers (government or private) and type of organization (national or multinational) with regard to breastfeeding breaks, breastfeeding corners, lighter jobs and paid maternity leave. Public and multinational companies were slightly better than private and national ones in providing breastfeeding facilities. | Ad hoc questionnaire | Pakistan |
| 39 | 2016 | Dagher R.K.; McGovern P.M.; Schold J.D.; Randall X.J. | Determinants of breastfeeding initiation and cessation among employed mothers: A prospective cohort study | BMC Pregnancy and Childbirth | Cohort study | 817 women aged 18 and older while hospitalized for childbirth. | The hazard for breastfeeding cessation by 6 months was higher for women who returned to work at any time during the 6 months postpartum versus those who did not return, lower for professional workers, higher among single than married women, higher for every educational category compared to graduate school, and higher for those with no family or friends who breastfeed. | Ad hoc questions administered in person and by phone, records from medical files | USA |
| 40 | 2016 | Desmond D.; Meaney S. | A qualitative study investigating the barriers to returning to work for breastfeeding mothers in Ireland | International Breastfeeding Journal | Qualitative study | 16 mothers who returned to the workforce. | Mothers with the desire to continue to breastfeed after their return to work did so with some difficulty. Many did not disclose to their employers that they were breastfeeding and did not make enquiries about being facilitated to continue to breastfeed after their return to the workplace. The perceived lack of support from their employers as well as embarrassment about their breastfeeding status meant many women concealed that they were breastfeeding after their return to the workplace. | Interviews | Ireland |
| 41 | 2016 | Lubold A.M. | Breastfeeding and employment: A propensity score matching approach | Sociological Spectrum | Cohort study | 746 women. | Mothers who return to paid work within three months of giving birth breastfeed an average of five fewer weeks than new mothers who do not return to paid work for three months or more. In addition, among mothers who return to paid work within three months, full-time workers breastfeed an average of more than 15 fewer weeks than part-timeworkers. | Ad hoc questionnaires, records from the Centers for Disease Control data | USA |
| 42 | 2016 | Spitzmueller C.; Wang Z.; Zhang J.; Thomas C.L.; Fisher G.G.; Matthews R.A.; Strathearn L. | Got milk? Workplace factors related to breastfeeding among working mothers | Journal of Organizational Behavior | Cohort study | 259 working mothers. | Women who return to work and continue breastfeeding experience more family-to-work conflict and overload than women who do not reconcile work and breast feeding. Breastfeeding goal intentions were negatively associated with the hazard rate of ceasing breast feeding after return to work (B= 0.16, Wald = 83.74, p<.001). | Ad hoc questionnaires and surveys | USA |
| 43 | 2016 | Xiang N.; Zadoroznyj M.; Tomaszewski W.; Martin B. | Timing of return to work and breastfeeding in Australia | Pediatrics | Cross-sectional study | 2,300 mothers in paid employment in the 13 months before giving birth. | Mothers who returned to work within 6 months and who worked for ≥20 hours per week were significantly less likely than mothers who had not returned to work to be breastfeeding at 6 months. However, returning to work for ≤19 hours per week had no significant impact on the likelihood of breastfeeding regardless of when mothers returned to work. Older maternal age, higher educational attainment, better physical or mental health, managerial or professional maternal occupation, and being self-employed all significantly contributed to the increased likelihood of any breastfeeding at 6 months. Similar patterns exist for predominant breastfeeding at 16 weeks. | Ad hoc survey | Australia |
| 44 | 2015 | Aikawa T, Pavadhgul P, Chongsuwat R, Sawasdivorn S, Boonshuyar C. | Maternal return to paid work and breastfeeding practices in Bangkok, Thailand | Asia Pac J Public Health | Cross-sectional study | 84 working mothers with a child aged 6 to 24 months. | Exclusive breastfeeding for 3 months was 78.6%, and for 6 months was 38.1%. Mothers who returned to work 3 months or more after giving birth exclusively breastfed more than the mothers who returned to work in less than 3 months (crude odds ratio [OR] = 4.26, 95% confidence interval [CI] = 1.39-13.05; adjusted OR = 4.15, 95% CI = 1.15-14.95). Moreover, mothers who worked at self-employed or family-owned businesses and some mothers working at private companies showed tendencies of returning to work in less than 3 months. | Ad hoc questionnaires | Thailand |
| 45 | 2015 | Bai D.L.; Fong D.Y.T.; Tarrant M. | Factors Associated with Breastfeeding Duration and Exclusivity in Mothers Returning to Paid Employment Postpartum | Maternal and Child Health Journal | Cohort study | 1,738 mothers who returned to paid employment postpartum | 32 % of the sample combined breastfeeding and employment. A later return to work and higher maternal education were associated with new mothers being able to combine breastfeeding and employment. Later return to work, shorter working hours, parental childcare, and higher maternal education were also associated with less likelihood of weaning from any or exclusive breastfeeding. | Ad hoc questionnaires | China |
| 46 | 2014 | Dagher R.K.; McGovern P.M.; Dowd B.E. | Maternity leave duration and postpartum mental and physical health: Implications for leave policies | Journal of Health Politics, Policy and Law | Cohort study | 716 employed women. | In the first postpartum year, an increase in leave duration is associated with a decrease in depressive symptoms until six months postpartum. Moreover, analysis showed a significant linear positive association between leave duration and physical health. | 1. Edinburgh Postnatal Depression Scale; 2. SF-12 Health Survey; 3. ad hoc questionnaire for maternal childbirth-related symptoms; 4. data from medical records. Data were collected from the Maternal Postpartum Health Study | USA |
| 47 | 2014 | Mandal B.; Roe B.E.; Fein S.B. | Work and breastfeeding decisions are jointly determined for higher socioeconomic status US mothers | Review of Economics of the Household | Cohort study | 2,236 women who worked before childbirth. | Returning to paid work 1 week earlier reduces any breastfeeding duration by about two-thirds of a week while extending breastfeeding by a week delays work participation by about one-third of a week. | Ad hoc surveys. Data were collected from the Infant Feeding Practices Study II (IFPS II) | USA |
| 48 | 2014 | Mirkovic K.R.; Perrine C.G.; Scanlon K.S.; Grummer-Strawn L.M. | Maternity leave duration and full-time/part-time work status are associated with us mothers' ability to meet breastfeeding intentions | Journal of Human Lactation | Cohort study | 1,172 women employed prenatally who intended to breastfeed 3 months or longer. | 28.8% of mothers did not meet their intention to breastfeed at least 3 months. Odds of not meeting intention to breastfeed at least 3 months were higher among mothers who returned to work full time before 3 months (< 6 weeks/full time: AOR = 2.25, 95% confidence interval, 1.23-4.12; 6 weeks-3 months/full time: AOR = 1.82, 95% confidence interval, 1.30-2.56), compared with mothers not working at 3 months. | Ad hoc surveys. Data were collected from the Infant Feeding Practices Study II (IFPS II) | USA |
| 49 | 2013 | Ahmadi M, Moosavi SM. | Evaluation of occupational factors on continuation of breastfeeding and formula initiation in employed mothers | Glob J Health Sci | Cross-sectional study | 212 mothers with infants aged 6-12 months. | 52.38% of the sample used formula to feed their children, and 27.36% had discontinued breastfeeding. The rate of formula use was significantly higher in mothers who had less than 6 months of maternity leave, those who did not have a suitable nursery or place to milk themselves and preserve the milk in their workplace, those working more than 6 hours per day, and those who could not take a breastfeeding break. | Ad hoc questionnaire | Iran |
| 50 | 2013 | Bonet M.; Marchand L.; Kaminski M.; Fohran A.; Betoko A.; Charles M.-A.; Blondel B. | Breastfeeding duration, social and occupational characteristics of mothers in the French 'EDEN mother-child' cohort | Maternal and Child Health Journal | Cohort study | 1,339 mothers who were breastfeeding at discharge from the maternity unit. | Returning to work was the major predictor for stopping breastfeeding: The sooner mothers returned to work, the less they breastfed their babies at 4 months, independently of full-time or part-time employment. | Ad hoc questionnaires, data from medical records. Data were collected from the Etude des Déterminants pré et postnatals précoces du développement et de la santé de l’Enfant (EDEN) study | France |
| 51 | 2013 | Parcsi L, Curtin M. | Experiences of occupational therapists returning to work after maternity leave | Aust Occup Ther J | Qualitative study | 6 occupational therapists. | Two major themes emerged: compromise and feeling valued. The experience of returning to work was a process of compromise in which women found strategies to cope with their changing roles and demands, to find a balance between home and work life. Women wanted to feel valued by their managers and co-workers, as this enabled them to feel comfortable and confident with some of the compromises they made. | Semi-structured interviews | Australia |
| 52 | 2013 | Tsai S.-Y. | Impact of a breastfeeding-friendly workplace on an employed mother's intention to continue breastfeeding after returning to work | Breastfeeding Medicine | Cross-sectional study | 715 working mothers. | Results underline that a higher education level (OR = 2.66), lower workload (8 work hours/day) (OR = 2.66), lactation room with dedicated space (OR = 2.38), use of breast pumping breaks (OR = 61.6), and encouragement from colleagues (OR = 2.78) and supervisors (OR = 2.44) to use breast pumping breaks were significant predictors of continued breastfeeding for more than 6 months after returning to work. | Ad hoc questionnaires | Taiwan |

**Table 2. Risk of Bias**

|  | **Authors** | **Item 1** | **Item 2** | **Item 3** | **Item 4** | **Item 5** | **Item 6** | **Item 7** | **Item 8** | **Item 9** | **Item 10** | **Item 11** | **Risk of Bias** |
| --- | --- | --- | --- | --- | --- | --- | --- | --- | --- | --- | --- | --- | --- |
| **Cross-sectional study** | Chen K.; Wei L.; Zhang Y.; Jiang W.; Wang J.; Pan Y. (2022) | · | · | · | · | · | · | · | · |  |  |  | Moderate |
|  | Jain S, Neaves S, Royston A, Huang I, Juengst SB. (2022) | · | · | · | · | · | · | · | · |  |  |  | Low |
|  | Jiravisitkul P.; Thonginnetra S.; Kasemlawan N.; Suntharayuth T. (2022) | · | · | · | · | · | · | · | · |  |  |  | Low |
|  | Tohme P, Abi-Habib R. (2022) | · | · | · | · | · | · | · | · |  |  |  | Low |
|  | Tsai S.-Y. (2022) | · | · | · | · | · | · | · | · |  |  |  | Moderate |
|  | Nguyen T.T.; Cashin J.; Tran H.T.T.; Vu D.H.; Nandi A.; Phan M.T.; Van N.D.C.; Weissman A.; Pham T.N.; Nguyen B.V.; Mathisen R. (2022) | · | · | · | · | · | · | · | · |  |  |  | Low |
|  | Ickes S.B.; Oddo V.M.; Sanders H.K.; Nduati R.; Denno D.M.; Myhre J.A.; Kinyua J.; Iannotti L.L.; Singa B.; Farquhar C.; Walson J.L. (2021) | · | · | · | · | · | · | · | · |  |  |  | Low |
|  | Butudom A.; McFarlin B.L.; Klima C.S.; Spatz D.L.; Kennelly J.F.; McCreary L.L.; Patil C.L.; Koenig M.D (2021) | · | · | · | · | · | · | · | · |  |  |  | Moderate |
|  | Cervera-Gasch Á.; Mena-Tudela D.; Leon-Larios F.; Felip-Galvan N.; Rochdi-Lahniche S.; Andreu-Pejó L.; González-Chordá V.M. (2020) | · | · | · | · | · | · | · | · |  |  |  | Low |
|  | Falletta L.; Abbruzzese S.; Fischbein R.; Shura R.; Eng A.; Alemagno S. (2020) | · | · | · | · | · | · | · | · |  |  |  | Low |
|  | Kebede T, Woldemichael K, Jarso H, Bekele BB. (2020) | · | · | · | · | · | · | · | · |  |  |  | Low |
|  | Abou-Elwafa H.S.; El-Gilany A.-H. (2019) | · | · | · | · | · | · | · | · |  |  |  | Moderate |
|  | Gianni M.L.; Bettinelli M.E.; Manfra P.; Sorrentino G.; Bezze E.; Plevani L.; Cavallaro G.; Raffaeli G.; Crippa B.L.; Colombo L.; Morniroli D.; Liotto N.; Roggero P.; Villamor E.; Marchisio P.; Mosca F. (2019) | · | · | · | · | · | · | · | · |  |  |  | Low |
|  | Juengst S.B.; Royston A.; Huang I.; Wright B. (2019) | · | · | · | · | · | · | · | · |  |  |  | High |
|  | Zhuang J.; Bresnahan M.J.; Yan X.; Zhu Y.; Goldbort J.; Bogdan-Lovis E. (2019) | · | · | · | · | · | · | · | · |  |  |  | Low |
|  | Hendaus M.A.; Alhammadi A.H.; Khan S.; Osman S.; Hamad A. (2018) | · | · | · | · | · | · | · | · |  |  |  | High |
|  | Horwood C.; Haskins L.; Engebretsen I.; Phakathi S.; Connolly C.; Coutsoudis A.; Spies L. (2018) | · | · | · | · | · | · | · | · |  |  |  | Moderate |
|  | Soomro JA, Shaikh ZN, Bijarani SA, Saheer TB. (2017) | · | · | · | · | · | · | · | · |  |  |  | Low |
|  | Xiang N.; Zadoroznyj M.; Tomaszewski W.; Martin B. (2016) | · | · | · | · | · | · | · | · |  |  |  | Moderate |
|  | Aikawa T, Pavadhgul P, Chongsuwat R, Sawasdivorn S, Boonshuyar C. (2015) | · | · | · | · | · | · | · | · |  |  |  | Low |
|  | Ahmadi M, Moosavi SM. (2013) | · | · | · | · | · | · | · | · |  |  |  | Low |
|  | Tsai S.-Y. (2013) | · | · | · | · | · | · | · | · |  |  |  | Moderate |
| **Cohort study** | Jones K.P.; Brady J.M.; Lindsey A.P.; Cortina L.M.; Major C.K. (2022) | · | · | · | · | · | · | · | · | · | · | · | Moderate |
|  | Castetbon K, Boudet-Berquier J, Salanave B. (2020) | · | · | · | · | · | · | · | · | · | · | · | Low |
|  | de Lauzon-Guillain B, Thierry X, Bois C, Bournez M, Davisse-Paturet C, Dufourg MN, Kersuzan C, Ksiazek E, Nicklaus S, Vicaire H, Wagner S, Lioret S, Charles MA. (2019) | · | · | · | · | · | · | · | · | · | · | · | Low |
|  | Kobayashi M.; Usui E. (2017) | · | · | · | · | · | · | · | · | · | · | · | high |
|  | Dagher R.K.; McGovern P.M.; Schold J.D.; Randall X.J. (2016) | · | · | · | · | · | · | · | · | · | · | · | Low |
|  | Lubold A.M. (2016) | · | · | · | · | · | · | · | · | · | · | · | Low |
|  | Spitzmueller C.; Wang Z.; Zhang J.; Thomas C.L.; Fisher G.G.; Matthews R.A.; Strathearn L. (2016) | · | · | · | · | · | · | · | · | · | · | · | Moderate |
|  | Bai D.L.; Fong D.Y.T.; Tarrant M. (2015) | · | · | · | · | · | · | · | · | · | · | · | Low |
|  | Dagher R.K.; McGovern P.M.; Dowd B.E. (2014) | · | · | · | · | · | · | · | · | · | · | · | Low |
|  | Mandal B.; Roe B.E.; Fein S.B. (2014) | · | · | · | · | · | · | · | · | · | · | · | Moderate |
|  | Mirkovic K.R.; Perrine C.G.; Scanlon K.S.; Grummer-Strawn L.M. (2014) | · | · | · | · | · | · | · | · | · | · | · | Moderate |
|  | Bonet M.; Marchand L.; Kaminski M.; Fohran A.; Betoko A.; Charles M.-A.; Blondel B. (2013) | · | · | · | · | · | · | · | · | · | · | · | Low |
| **Qualitative study** | Oh E.; Mun E. (2022) | · | · | · | · | · | · | · | · | · | · |  | Low |
|  | Gebrekidan K.; Plummer V.; Fooladi E.; Hall H. (2021) | · | · | · | · | · | · | · | · | · | · |  | Low |
|  | Gregory S.K. (2021) | · | · | · | · | · | · | · | · | · | · |  | Moderate |
|  | Ickes S.B.; Sanders H.; Denno D.M.; Myhre J.A.; Kinyua J.; Singa B.; Lemein H.S.; Iannotti L.L.; Farquhar C.; Walson J.L.; Nduati R. (2021) | · | · | · | · | · | · | · | · | · | · |  | Low |
|  | Moulton K.L.; Battaglioli N.; Sebok-Syer S.S. (2021) | · | · | · | · | · | · | · | · | · | · |  | Moderate |
|  | Wolde FB, Ali JH, Mengistu YG. (2021) | · | · | · | · | · | · | · | · | · | · |  | Low |
|  | Hasan A.M.R.; Smith G.; Selim M.A.; Akter S.; Khan N.U.Z.; Sharmin T.; Rasheed S. (2020) | · | · | · | · | · | · | · | · | · | · |  | Low |
|  | Mabaso B.P.; Jaga A.; Doherty T. (2020) | · | · | · | · | · | · | · | · | · | · |  | Low |
|  | Trafford Z.; Jewett S.; Swartz A.; Lefevre A.E.; Winch P.J.; Colvin C.J.; Barron P.; Bamford L. (2020) | · | · | · | · | · | · | · | · | · | · |  | Low |
|  | Burns E, Triandafilidis Z. (2019) | · | · | · | · | · | · | · | · | · | · |  | Moderate |
|  | Dal Forno Martins G.; Leal C.L.; Schmidt B.; Piccinini C.A. (2019) | · | · | · | · | · | · | · | · | · | · |  | Low |
|  | Febrianingtyas Y.; Februhartanty J.; Hadihardjono D.N. (2019) | · | · | · | · | · | · | · | · | · | · |  | Low |
|  | Riaz S, Condon L. (2019) | · | · | · | · | · | · | · | · | · | · |  | Moderate |
|  | Sulaiman Z, Liamputtong P, Amir LH. (2018) | · | · | · | · | · | · | · | · | · | · |  | Low |
|  | Zhang Y.; Jin Y.; Vereijken C.; Stahl B.; Jiang H. (2018) | · | · | · | · | · | · | · | · | · | · |  | Low |
|  | Hmone M.P.; Li M.; Agho K.; Alam A.; Dibley M.J. (2017) | · | · | · | · | · | · | · | · | · | · |  | Low |
|  | Desmond D.; Meaney S. (2016) | · | · | · | · | · | · | · | · | · | · |  | Moderate |
|  | Parcsi L, Curtin M. (2013) | · | · | · | · | · | · | · | · | · | · |  | Moderate |
|  | *Color code: Yes=·; No=·; Unclear=·; Not applicable=·* |  |  |  |  |  |  |  |  |  |  |  |  |
|  |  |  |  |  |  |  |  |  |  |  |  |  |  |
| *Cross sectional* |  |  |  |  |  |  |  |  |  |  |  |  |  |
| JBI Q1 | Were the criteria for inclusion in the sample clearly defined? |  |  |  |  |  |  |  |  |  |  |  |  |
| JBI Q2 | Were the study subjects and the setting described in detail? |  |  |  |  |  |  |  |  |  |  |  |  |
| JBI Q3 | Was the exposure measured in a valid and reliable way? |  |  |  |  |  |  |  |  |  |  |  |  |
| JBI Q4 | Were objective, standard criteria used for measurement of the condition? |  |  |  |  |  |  |  |  |  |  |  |  |
| JBI Q5 | Were confounding factors identified? |  |  |  |  |  |  |  |  |  |  |  |  |
| JBI Q6 | Were strategies to deal with confounding factors stated? |  |  |  |  |  |  |  |  |  |  |  |  |
| JBI Q7 | Were the outcomes measured in a valid and reliable way? |  |  |  |  |  |  |  |  |  |  |  |  |
| JBI Q8 | Was appropriate statistical analysis used? |  |  |  |  |  |  |  |  |  |  |  |  |
|  |  |  |  |  |  |  |  |  |  |  |  |  |  |
| *Cohort study* |  |  |  |  |  |  |  |  |  |  |  |  |  |
| JBI Q1 | Were the two groups similar and recruited from the same population? |  |  |  |  |  |  |  |  |  |  |  |  |
| JBI Q2 | Were the exposures measured similarly to assign people to both exposed and unexposed groups? |  |  |  |  |  |  |  |  |  |  |  |  |
| JBI Q3 | Was the exposure measured in a valid and reliable way? |  |  |  |  |  |  |  |  |  |  |  |  |
| JBI Q4 | Were confounding factors identified? |  |  |  |  |  |  |  |  |  |  |  |  |
| JBI Q5 | Were strategies to deal with confounding factors stated? |  |  |  |  |  |  |  |  |  |  |  |  |
| JBI Q6 | Were the groups/participants free of the outcome at the start of the study (or at the moment of exposure)? |  |  |  |  |  |  |  |  |  |  |  |  |
| JBI Q7 | Were the outcomes measured in a valid and reliable way? |  |  |  |  |  |  |  |  |  |  |  |  |
| JBI Q8 | Was the follow up time reported and sufficient to be long enough for outcomes to occur? |  |  |  |  |  |  |  |  |  |  |  |  |
| JBI Q9 | Was follow up complete, and if not, were the reasons to loss to follow up described and explored? |  |  |  |  |  |  |  |  |  |  |  |  |
| JBI Q10 | Were strategies to address incomplete follow up utilized? |  |  |  |  |  |  |  |  |  |  |  |  |
| JBI Q11 | Was appropriate statistical analysis used? |  |  |  |  |  |  |  |  |  |  |  |  |
|  |  |  |  |  |  |  |  |  |  |  |  |  |  |
| *Case control* |  |  |  |  |  |  |  |  |  |  |  |  |  |
| JBI Q1 | Were the groups comparable other than the presence of disease in cases or the absence of disease in controls? |  |  |  |  |  |  |  |  |  |  |  |  |
| JBI Q2 | Were cases and controls matched appropriately? |  |  |  |  |  |  |  |  |  |  |  |  |
| JBI Q3 | Were the same criteria used for identification of cases and controls? |  |  |  |  |  |  |  |  |  |  |  |  |
| JBI Q4 | Was exposure measured in a standard, valid and reliable way? |  |  |  |  |  |  |  |  |  |  |  |  |
| JBI Q5 | Was exposure measured in the same way for cases and controls? |  |  |  |  |  |  |  |  |  |  |  |  |
| JBI Q6 | Were confounding factors identified? |  |  |  |  |  |  |  |  |  |  |  |  |
| JBI Q7 | Were strategies to deal with confounding factors stated? |  |  |  |  |  |  |  |  |  |  |  |  |
| JBI Q8 | Were outcomes assessed in a standard, valid and reliable way for cases and controls? |  |  |  |  |  |  |  |  |  |  |  |  |
| JBI Q9 | Was the exposure period of interest long enough to be meaningful? |  |  |  |  |  |  |  |  |  |  |  |  |
| JBI Q10 | Was appropriate statistical analysis used? |  |  |  |  |  |  |  |  |  |  |  |  |
|  |  |  |  |  |  |  |  |  |  |  |  |  |  |
| *Qualitative study* |  |  |  |  |  |  |  |  |  |  |  |  |  |
| JBI Q1 | Is there congruity between the stated philosophical perspective and the research methodology | | | | | |  |  |  |  |  |  |  |
| JBI Q2 | Is there congruity between the research methodology and the research question or objectives | | | | | |  |  |  |  |  |  |  |
| JBI Q3 | Is there congruity between the research methodology and the methods used to collect data? | | | | |  |  |  |  |  |  |  |  |
| JBI Q4 | Is there congruity between the research methodology and the representation and analysis of data? | | | | | |  |  |  |  |  |  |  |
| JBI Q5 | Is there congruity between the research methodology and the interpretation of results? | | | | |  |  |  |  |  |  |  |  |
| JBI Q6 | Is there a statement locating the researcher culturally or theoretically? | | |  |  |  |  |  |  |  |  |  |  |
| JBI Q7 | Is the influence of the researcher on the research, and vice- versa, addressed? | | |  |  |  |  |  |  |  |  |  |  |
| JBI Q8 | Are participants, and their voices, adequately represented? |  |  |  |  |  |  |  |  |  |  |  |  |
| JBI Q9 | Is the research ethical according to current criteria or, for recent studies, and is there evidence of ethical approval by an appropriate body? | | | | | | | | | | |  |  |
| JBI Q10 | Do the conclusions drawn in the research report flow from the analysis, or interpretation, of the data? | | | | | | |  |  |  |  |  |  |

**Table 3. *PRISMA Checklist***

| **Section and Topic** | **Item #** | **Checklist item** | **Location where item is reported** |
| --- | --- | --- | --- |
| **TITLE** | | |  |
| Title | 1 | Identify the report as a systematic review. | Title |
| **ABSTRACT** | | |  |
| Abstract | 2 | See the PRISMA 2020 for Abstracts checklist. | Page 1 |
| **INTRODUCTION** | | |  |
| Rationale | 3 | Describe the rationale for the review in the context of existing knowledge. | Pages 2-3 |
| Objectives | 4 | Provide an explicit statement of the objective(s) or question(s) the review addresses. | Page 3 |
| **METHODS** | | |  |
| Eligibility criteria | 5 | Specify the inclusion and exclusion criteria for the review and how studies were grouped for the syntheses. | Page 4-5 and Figure 1 |
| Information sources | 6 | Specify all databases, registers, websites, organisations, reference lists and other sources searched or consulted to identify studies. Specify the date when each source was last searched or consulted. | Pages 3-5 |
| Search strategy | 7 | Present the full search strategies for all databases, registers and websites, including any filters and limits used. | Figure 1 |
| Selection process | 8 | Specify the methods used to decide whether a study met the inclusion criteria of the review, including how many reviewers screened each record and each report retrieved, whether they worked independently, and if applicable, details of automation tools used in the process. | Pages 4-5 |
| Data collection process | 9 | Specify the methods used to collect data from reports, including how many reviewers collected data from each report, whether they worked independently, any processes for obtaining or confirming data from study investigators, and if applicable, details of automation tools used in the process. | Page 7 |
| Data items | 10a | List and define all outcomes for which data were sought. Specify whether all results that were compatible with each outcome domain in each study were sought (e.g. for all measures, time points, analyses), and if not, the methods used to decide which results to collect. | Supp. table 1 |
|  | 10b | List and define all other variables for which data were sought (e.g. participant and intervention characteristics, funding sources). Describe any assumptions made about any missing or unclear information. | Supp. table 1 |
| Study risk of bias assessment | 11 | Specify the methods used to assess risk of bias in the included studies, including details of the tool(s) used, how many reviewers assessed each study and whether they worked independently, and if applicable, details of automation tools used in the process. | Page 5 and Supp. Table 2 |
| Effect measures | 12 | Specify for each outcome the effect measure(s) (e.g. risk ratio, mean difference) used in the synthesis or presentation of results. | Pages 6-8 |
| Synthesis methods | 13a | Describe the processes used to decide which studies were eligible for each synthesis (e.g. tabulating the study intervention characteristics and comparing against the planned groups for each synthesis (item #5)). | Pages 6-8 |
|  | 13b | Describe any methods required to prepare the data for presentation or synthesis, such as handling of missing summary statistics, or data conversions. | Pages 7-8 |
|  | 13c | Describe any methods used to tabulate or visually display results of individual studies and syntheses. | Page 5 and Supp. Table 1. |
|  | 13d | Describe any methods used to synthesize results and provide a rationale for the choice(s). If meta-analysis was performed, describe the model(s), method(s) to identify the presence and extent of statistical heterogeneity, and software package(s) used. | Page 5 |
|  | 13e | Describe any methods used to explore possible causes of heterogeneity among study results (e.g. subgroup analysis, meta-regression). | n/a |
|  | 13f | Describe any sensitivity analyses conducted to assess robustness of the synthesized results. | n/a |
| Reporting bias assessment | 14 | Describe any methods used to assess risk of bias due to missing results in a synthesis (arising from reporting biases). | n/a |
| Certainty assessment | 15 | Describe any methods used to assess certainty (or confidence) in the body of evidence for an outcome. | n/a |
| **RESULTS** | | |  |
| Study selection | 16a | Describe the results of the search and selection process, from the number of records identified in the search to the number of studies included in the review, ideally using a flow diagram. | Figure 1 |
|  | 16b | Cite studies that might appear to meet the inclusion criteria, but which were excluded, and explain why they were excluded. | n/a |
| Study characteristics | 17 | Cite each included study and present its characteristics. | Supp. Table 1 |
| Risk of bias in studies | 18 | Present assessments of risk of bias for each included study. | Supp. Table 2 |
| Results of individual studies | 19 | For all outcomes, present, for each study: (a) summary statistics for each group (where appropriate) and (b) an effect estimate and its precision (e.g. confidence/credible interval), ideally using structured tables or plots. | Supp. Table 1 |
| Results of syntheses | 20a | For each synthesis, briefly summarise the characteristics and risk of bias among contributing studies. | n/a |
|  | 20b | Present results of all statistical syntheses conducted. If meta-analysis was done, present for each the summary estimate and its precision (e.g. confidence/credible interval) and measures of statistical heterogeneity. If comparing groups, describe the direction of the effect. | Pages 6-8 |
|  | 20c | Present results of all investigations of possible causes of heterogeneity among study results. | n/a |
|  | 20d | Present results of all sensitivity analyses conducted to assess the robustness of the synthesized results. | n/a |
| Reporting biases | 21 | Present assessments of risk of bias due to missing results (arising from reporting biases) for each synthesis assessed. | n/a |
| Certainty of evidence | 22 | Present assessments of certainty (or confidence) in the body of evidence for each outcome assessed. | n/a |
| **DISCUSSION** | | |  |
| Discussion | 23a | Provide a general interpretation of the results in the context of other evidence. | Pages 13-16 |
|  | 23b | Discuss any limitations of the evidence included in the review. | Page 16 |
|  | 23c | Discuss any limitations of the review processes used. | Page 16 |
|  | 23d | Discuss implications of the results for practice, policy, and future research. | Pages 16-17 |
| **OTHER INFORMATION** | | |  |
| Registration and protocol | 24a | Provide registration information for the review, including register name and registration number, or state that the review was not registered. | n/a |
|  | 24b | Indicate where the review protocol can be accessed, or state that a protocol was not prepared. | n/a |
|  | 24c | Describe and explain any amendments to information provided at registration or in the protocol. | n/a |
| Support | 25 | Describe sources of financial or non-financial support for the review, and the role of the funders or sponsors in the review. | n/a |
| Competing interests | 26 | Declare any competing interests of review authors. | n/a |
| Availability of data, code and other materials | 27 | Report which of the following are publicly available and where they can be found: template data collection forms; data extracted from included studies; data used for all analyses; analytic code; any other materials used in the review. | n/a |
